# Abstracts and Gleanings

**Published:** 1883-02-20

**Authors:** 


### ABSTRACTS AND GLEANINGS.

**Bromide of Ethyl, the Most Perfect Anæsthetic for Short, Painful Surgical Operations.**—Prof. Chisolm, in a paper read before the Academy of Medicine, Baltimore, after confessing former ill success with this agent and a consequent prejudice against it, says: "Having found out how to use it, and what to expect from its administration, I can obtain the most brilliant results from it, and have become quite enthusiastic in its praises.

"In every patient, using the needful precaution, I have produced complete narcosis in less than one minute, often in from twenty to thirty seconds. A deep sleep which, however, will not last more than one or two minutes. From this speedily induced narcosis recovery is rapid and complete, with neither nausea nor heaviness, so that, as a rule, five minutes after the inhalation the patient is as much himself as if no anæsthetic had been used. Experience has taught me that these are the peculiarities of the bromide of ethyl when administered for anæsthetic purposes, and that as such they will prove of inestimable value to surgery.

"The following very interesting cases, patients recently operated upon, will illustrate how thoroughly and speedily the brain resumes its full function after complete ethyl narcosis:

"Miss M., a self-possessed little girl, eight years of age, desired to have an ugly squint corrected, and exhibited no timidity in witnessing the preparations needful for its performance. Prior to getting upon the table she had her collar loosened to remove any impediment to respiration. In doing so she took two roses from her dress and placed them on a vacant chair near by. She was then put on the operating table and the bromide of ethyl administered. A very few inspirations produced deep sleep, under which the tenotomy of the rectus muscle was performed. The ethylization and squint operation occupied fifty-six seconds: the time was taken by one of my assistants. Within three minutes from the commencement of the narcotism the child was perfectly awake, and was ready to get from the table. When on the floor she walked at once to the chair, and within four minutes from the time that the anæsthesia was commenced she was engaged in pinning these roses into the front of her dress, with a composure which showed not only no present discomfort, but a complete oblivion of the experience through which she had just passed.

"The second case, also one of convergent squint, was that of a boy, fifteen years of age, who seemed very anxious to get rid of his deformity. After getting on the operating table, before the medical class at the University of Maryland clinic, I told him that when the towel was placed over his face it would have a very choking sensation, but that he could not choke from it. I also showed him how to take quick and full inspirations, so that the suffocative sensations would entirely pass away before he had breathed a half dozen times. When the folded towel, upon which a drachm of ethyl had been poured, was placed over his face he commenced a most active respiratory movement, which, in a very few seconds, quieted down into deep sleep. Within thirty seconds from the commencement of the ethylization narcotism was profound. The operation was commenced without delay and the division of the tendon speedily consummated. The entire operation, from the commencing ethylization to the perfection of the tenotomy, did not exceed sixty seconds. A minute had not elapsed from the completion of the operation when he awoke, and, jumping from the table to the floor of the amphitheatre, he cried out in a jubilant voice, 'I am all right,' much to the amusement of the medical class who crowded the benches: a very different behavior from that which follows the inhalation of chloroform or ether. In this case the entire period, from the beginning of the inhalation, through the stage of complete narcosis, to perfect restoration, did not exceed two minutes."

Other cases are given with like results. He further remarks:

"Experience, by daily administration, has taught me this very valuable lesson, viz., that the Bromide of Ethyl is not an anæsthetic which can be advantageously repeated, or its inhalation be continued for any length of time. This is one of the serious mistakes which we made in our early experiments, and which induced me, through ignorance, to discard the new agent as unreliable.

"Its wonderful action is obtained during the first minute of its inhalation, and what I have called its primary anæsthesia.

"In cases in which, from some interference with the rapidity of the manual of operative procedure, this primary anæst*h*esia wears off, and a second, and even more numerous administrations have to be made to keep up the anæsthetic state until the operation can he completed, while the narcosis can at all times be reproduced, nausea is very apt to follow. By this frequent repetition of the inhalation, a mental depression is established, as from the continued use of chloroform or ether, which may last many hours.

"Fortunately, there are many surgical operations of a very painful nature which can be perfected within the short period of a primary ethyl narcosis. Abscesses can be lanced, cysts emptied, sinuses laid open, wounds probed, strictures incised, muscles divided, ingrowing nails removed, surfaces cauterized, examinations made necessitating painful manipulations, and even amputations may be performed. It must not be forgotten that prior to the discovery of anæsthetics, Mr. Liston urged the general adoption of flap amputations, because all painful cutting, including the sawing of the bones, could be completed in so many seconds, and did not require minutes, at the hands of dextrous surgeons.

"To use the bromide of ethyl efficiently, one must have confidence in himself and, also, in the safety of the agent which he is administering.

"For long operations, or such as I desire to complete slowly, I prefer to administer chloroform, an anæsthetic with which I have had a long, extensive and uninterruptedly satisfactory experience. Of over twelve thousand patients upon whom I have operated under the narcotic effects of chloroform, I have not lost one. These patients cover organic disorders of heart, lungs, kidney or visceral disease, in persons of all ages, from the child only a few days old to my oldest chloroform administration, a very old man of ninety-six. Some were strong while others were very feeble. I never refuse the comforts of an anæsthetic to any person upon whom I have to operate.

We now append Dr. Chisolm's method of using chloroform:

"1. I always, without a single exception, give a strong drink of whiskey, from one to two ounces, to every adult to whom I intend to administer chloroform. This is done a few minutes before they get on the operating table. Because I never omit this fundamental law, and in advance sustain the heart against the depressing effect of the anæsthetic, in not one of my twelve thousand cases have I ever had to use, in a single instance, a hypodermic of whiskey. It is already in the stomach should it be needed, and can do no harm if not required.

"2. Always loose the neck and chest clothing, so as to have no impediment to respiration.

"3. Only administer chloroform in the recumbent posture, with body perfectly horizontal and head on a low pillow, this pillow to be removed as the anæsthesia progresses.

"4. Give chloroform on a thin towel folded in conical form with open apex, so that the vapor, before inhalation, will be freely diluted with atmospheric air. In holding this cone over the face of the patient at some little distance from the nose, place the fingers under the borders of the cone for the double purpose of allowing air to enter freely and, also, to prevent the chloroform liquid on the towel from coming in contact with the skin of the patient's face, and thereby avoid its blistering effects.

"5. Should loud snoring occur, force up the chin. This manipulation, by straightening the air passages from the nose to the larynx, makes easy breathing. The forcible elevation of the chin is far better in every respect than pulling out the tongue. It is easier of application, more quickly done, requires no instruments, and is much more efficient in removing the impediment to respiration.

"By always following these five simple rules, I have had, so far, both safety and eomfort in the administration of chloroform.

"Possibly, one very strong reason why I have becn so successful in the administration of chloroform is that, as a specialist in eye surgery, the inhaler must be removed from the nose before I commence the surgical manipulations. Besides, while operating, I have constantly in view both the color of the face and the respiration of the patient, which I consider even more important for the surgeon to observe than to feel the pulse. When surgeons are operating on distant parts of the body and cannot watch the work of the administrator of chloroform, accidents are most apt to happcn.

"In the inhalation of the bromide of ethyl, all of these rules laid down for the establishing of chloroform are not necessary, and some of them cannot be followed out.

"The recumbent posture I consider essential for the safe administration of any anæsthetic, whether it be chloroform, ether or ethyl; hence, these agents are not safe remedies at the hands of dentists, who place their patients in a sitting posture. Preparatory to the inhalation of the bromide of ethyl, I have not found it necessary to give whiskey. The only precaution I take is to loose the neck clothing and have the patient lie down with the head only slightly elevated."

### His Method of Administering the Bromide of Ethyl.

"My experiments have taught me that the mode of administering the ethyl should differ totally from that used in giving chloroform.

"Instead of a chloroform vapor freely diluted with atmospheric air, a saturated ethyl vapor must be inhaled, to the exclusion of atmospheric air, in order to obtain speedily and effectually narcosis.

"In my early experiments with this new agent I had not yet discovered this fundamental principle, and hence did not obtain good results. I voted bromide of ethyl a failure, because in common with other experimenters, I was too timid, or, rather, I should say, too ignorant of its peculiarities, to push the ethyl vapor in the concentrated form, which I have since found necessary to obtain good results. By my present method of administering it, I can obtain perfect ethylization in patients in from twenty to sixty seconds, and have no after consequences of nausea or dullness of feeling.

The best inhaler for the giving of the bromide of ethyl is a thick towel folded into the form of a small cone with closed apex. Between one of the folds of the towel I place a sheet of paper, which makes the cone nearly air tight. The base of the cone must be wide enough to enclose both mouth and nose. The soft material of which the inhaler is made enables the rim to be kept firmly in contact with the face, so as to exclude air from entering. I always instruct the patient how to make long inspirations, and inform him that he must do this, notwithstanding the fact that he will feel somewhat stifled. I also try to give him confidence by assuring him that a very few inspirations will put him to sleep. Usually I make him go through the process of strong respiratory movements in advance, so that he will know exactly how to proceed. Into this towel cone I pour about one drachm of the bromide of ethyl and immediately invert the inhaler over the nose and mouth of the patient, holding its edge down firmly over the face. There is no fear of creating asphyxia, as all air cannot be excluded, and the height of the cone makes a considerable air chamber into which the patient breathes.

Children usually struggle to escape from the apparatus. The cone, however, must not be removed from the face for an instant until anæsthesia is produced. At first some patients will resist the breathing of the vapor, but there is no fear that they will not catch their breath in time. Should children cry, it only insures inspiratory efforts, which the more surely and quickly will bring about the introduction of the vapor into the lungs. As a rule, a dozen full inspirations are all that are needed to produce deep narcosis. I recognize this desirable condition by a stoppage of all struggling. I have had deep sleep brought on by the sixth inspiration, when complete relaxation ensues, with quiet breathing, and an absence of reflex irritation should the conjunctiva be touched. The patient retains the usual healthy color of lips and cheeks, as if in ordinary sleep, and the pulse becomes slower and stronger as the narcosis becomes profound. Thirty seconds, as a rule, is sufficient to bring about this desirable condition, and have the patient ready for operation.

I have not found this anæsthetic sleep last more than two or three minutes, ofton not so long.

Usually the patients awake suddenly, and as completely as they would do from ordinary sleep. They are able to get down from the operating table without assistance and walk of without staggering, and with brain clear to answer correctly any question: in fact, quite themselves.

It took me some time to acquire such confidence in the safety of the remedy as to apply it in the concentrated form needful to obtain its fullest benefits. To the uninitiated it looks like cruel work to keep the cone of a saturated ethylized vapor over the face of a struggling patient. I am convinced, however, that in no other way can quick, complete and safe anæsthesia be obtained by it. Fortunately the struggling is very soon over, and quiet sleep speedily ensues.

My experience with the bromide of ethyl will now exceed four hundred cases, of which upward of three hundred are within the past year. I am beginning to be familiar with its administration and its effects. I now know what is to be obtained by it, and what not to expect from. I give it without hesitation, in any case, to avoid painful manipulation. I have used it as often as six times a day, and I administer it, on an average, certainly once every day. In the last week I have given it fifteen times. For office use I find it invaluable, on account of its promptness, efficiency, evanescent nature of the anæsthesia induced, the absence of nausea, and the perfect comfort with which patients operated upon can leave my office within a few minutes after the ethylization. Its use in my every day experience does not interfere with the routine of office practice, nor occupy more time than I give to an ordinary office consultation, a very important desideratum to those who have restless patients awaiting their turn in the reception room.

Those who will use it by a single inhalation to produce a short, deep sleep, and not resort to a mal-administration of this very valuable, powerful agent for a continued anæsthesia, which it is incapable of sustaining in safety and in comfort, will become as enthusiastic as I am over its brilliant results. They will, in time, learn to consider it, as I do, the most perfect of anæsthetic agents for quick, painful surgical work. It can never take the place of chloroform or sulphuric ether where any heavy operations are to be done. These well-known and tried anæsthetics must continue in favor for all tedious operations, and will be used in minor surgery by those who manipulate slowly and who do not have prompt, quick assistants. But when one can take advantage of a primary anæsthesia from the first administration of the bromide of ethyl, and, having made every preparation in advance, will manipulate quickly, the new anæsthetic leaves nothing to be desired.

I will repeat, "can anything be more brilliant in surgery than a successful operation for squint, where an ugly deformity of years standing is promptly, thoroughly, safely and surely removed in less than one minute of time—fifty-two seconds for ethylization and operation?" This is the nearest approach to magic in the art of surgery.

**The Acceleration of Delivery in Peurperal Convulsions.**— The Medical Times and Gazette says that a recent number of the Achiv fur Gynaokologie contains an article entitled "A Contribution to our Knowledge of Eclampsia," by Dr. Fr. Schauta, assistant in the clinic of Professor Spath,of Vienna. The paper gives statistics based upon the large number of 134,345 labors, among which 344 cases of convulsions occurred. Figures are furnished bearing upon many points in the natural history of this disease, which are of much value, and deserve the attention of specialists. We purpose only, however, here to call the attention of our readers to the important practical point which is indicated in the title of these remarks.

Convulsions coming on during pregnancy quite as often, according to Dr. Schauta, persist during labor, as cease before that process begins. Out of 42 such cases, in 22 the fits continued to occur during labor, while in 20 they abated before its commencement.

The commonly received opinion that convulsions first attacking the patient during labor, commonly cease when delivery is complete, Dr. Schauta finds to a great extent negatived by the facts he has collected. Out of 185 cases in which convulsions came on during labor, in only 62 were they limited to this period, while in 123 they continued after the patient had been delivered, *i. e.*, in 66·5 per cent. In 38 of these the frequency of the attacks postpartum was diminished, but 50 was increased; in the others there was no particular difference. Dr. Schauta quotes Brummerstadt, who had before him brought out the same fact. Out of 63 cases collected by the latter author, in only 18 did the attacks cease after delivery, in 17 they became less severe, and remained apparently uninfluenced in 28. The figures, therefore, both of Schauta and Brummerstadt seem to show that delivery does not, as a rule, exert a favorable influence upon peurperal convulsions.

The practical point, says Dr. Schauta, which springs out of these figures seems to be this—that in labor complicated with convulsions, the accoucheur should not allow himself to be pursuaded into operative delivery unless the clearest indications exist, and the necessary conditions are present; and that *accouchement force*, now on other grounds rightly abandoned, should, looking at the prognosis or peurperal eclampsia, be unconditionally condemned. Our author proceeds to test this practical conclusion by analyzing the cases according to whether or not labor was artificially completed. Out of the 42 cases of convulsions occurring during pregnancy, 20 were delivered spontaneously, 21 by the help of the accoucheur

(one passed from observation undelivered). Of the former, 2 died, or 10 per cent,; of the latter, 19, or 90.4 per cent. This nineteen, however, includes 5 who were delivered by Cæsarian section after the death of the mother; the subtraction of these reduces the mortality to 87.5 per cent. Of course it will be obvious that the cases in which interference to effect delivery was resorted to, were probably the worst cases, and the enormous difference in the result between those left to nature and those artificially delivered, is probably for the most part to be accounted for in this way. But admitting this, it is also evident that the acceleration of labor did not do very much for the patients. The result shown by cases of convulsions coming on during labor is much the same. Out of 185 cases, 53 were delivered by the natural efforts, and of these 14 died, or 26.4 per cent.; while of 132 who were delivered by obstetric aid, 54 died, or 40.9 per cent. With reference to these results, we again quote Dr. Schauta's remarks verbatim. He says: "In consequence of what has been said, the charge may readily be imputed to me of wishing to totally condemn all operative interference in puerperal eclampsia. I do not permit myself to draw so sweeping a conclusion from my statistics; but with reference to the sanguine expectations at present in the ascendent as to the effect of delivery in eclampsia, I may point to them as showing how little these hopes are fulfilled by the facts." There are two good reasons our author points out, for hastening delivery—if we can do it without harm. The first is, that by emptying the uterus, the intra-abdominal pressure, which in the large majority of cases is a main cause of the kidney-changes which produce eclampsia, is reduced, and therefore the earlier delivery takes place the *restitutio ad norman* may be expected to begin. The second is, that the sooner delivery is effected, the better chance the child has of survival. The risk to the child, as well as the mother, Dr. Schuta shows, is in proportion to the number of fits. The prognosis for the child is, as perhaps might be expected, worse when the fits come on before, than when they commence during labor. The infantile mortality among the cases of the former class which Dr. Schauta tabulates was 41.8 per cent.; among those of the latter 50.5 per cent.

In considering, in the light of these figures, whether in eclampsia delivery ought to be hastened, the question naturally occurs, whether the bad results enumerated may not have been the result of an aggravation of the nervous condition by the operation necessary to effect delivery, *i. e.*, whether operative delivery *per se* has any influence in producing convulsions. Dr. Schauta has, with this point in mind, analyzed the cases in which eclampsia appeared after labor. He finds that 74 of these had been naturally delivered, of whom 19 died, or 25.6 per cent.; 8 had been delivered by opative aid, of whom 2 died, or 25 per cent.—a proportion nearly the same.

These figures seem to us of considerable practical moment. It would be going too far to regard the high mortality among those who were delivered by art as due solely to the mere fact of interference. It seems to us largely explained by the consideration that the cases in which this treatment was resorted to were probably the worst, and it also has been sometimes the case that the state of the patient led the medical attendant to hurry delivery more than he would have done had death seemed less imminent, and in doing so to inflict damage which might have been avoided had less haste been used. If pregnancy has anything to do with the causation of the disease in question—and that it has, we think, there cannot be a doubt—we might expect that the removal of so powerful a cause would favor recovery. But Dr. Schauta's cases show this—that there is no such immediate advantage as to justify us in running any risk of other dangers for the sake of speedily ending the pregnancy. If labor has begun, or if it has been induced, it is best left to its course with the minimum of interference. It seems to us still an open question whether labor may not be induced with advantage, provided the process be conducted in a manner as closely as possible approximating to that of nature; but whether induced or at the natural term, such interference as would be called for if there were no convulsions is alone that which is required. Everything further is submitting the patient to unnecessary risk, without any compensating advantage.—*Quarterly Compendium of Medical Science.*

**Hemorrhagic Malarial Fever.**—In a well marked case of this affection, reported in Southern Practitioner, by Dr. Joiner, of Andersonville, Ga., the following was the treatment reported:

He was afraid of mercury, had lost confidence in quinine, distrusted turpentine, dispensed with blisters, and condemned opiates. Something must be done—and quickly. But, what? We feel sure that the kidneys are in trouble, but what is the nature of that trouble? Are they sick, intoxicated, asleep, or are they heroically endeavoring to respond to an unreasonable and impossible call upon them? We do not know.

In this trying dilemma we decided to appeal to the skin for help without delay, and to be governed by the circumstances as to treatment hereafter. We directed the patient to swallow as much hot water as he could, only a few ounces at first, repeated frequently in larger quantities, which course soon brought about freer and much easier discharges from the stomach, with a marked temporary quieting of nausea. At the same time, we directed bottles of hot water placed around about him. This was done and we anxiously watched for results.

In less than an hour our patient was bathed in a profuse perspiration; pulse 110; respiration 24; stomach comparatively quiet, and feeling every way decidedly more comfortable. For the first time we began to feel hopeful. The skin had nobly answered to the call made, and was casting off large quantities of liquid, heavily loaded with bilious matter, taking from the blood injurious and effete substances, giving the liver and kidneys that assistance so very necessary at this particular juncture. We could but feel anxiously hopeful.

Anticipating a chill in the afternoon, we ordered quinine 40 grains, divided into four powders, and to be given every two hours. We now placed on tongue one grain hydrag chlor. mite., and retired. At 5 P. M., on our return, found patient doing well. Pulse 100, respiration 24, nausea still less distressing, though less troublesome. No discharge of urine since morning. Ordered quinine continued in three-grain doses every three hours. Directed:

℞ Chlor. potass..................................ʒi

Aquæ........................................F ℥ij

M. Et. ft. sol.

Sig. Tablespoonful every three hours.

Ordered turpentine stupes to back, immediately over kidneys, and mush poultices thoroughly saturated with turpentine to stomach.

We left our patient still bathed in profuse perspiration and comparatively quiet.

At 9 P. M. we found our patient about as we had left him, save that he was suffering somewhat more from nausea. We now administered hypodermically one-fourth grain sulphate morphia. Continued treatment.

At 8 A. M. next day called, and learned that he had passed a fair night, having only suffered occasionally from nausea, and that he had slept at least one-half the time. Pulse now 100, respiration 24. No change in appearance of skin, save that it had assumed, perhaps, a deeper yellow color. Continued turpentine stupes to back, and poultices to stomach. Discontinued "potash," and ordered:

℞ Phos. acid......................................ʒi

Mur. tinc. iron..............................

Spts. lemon, aa..............................ʒij

Simp. syrup..................................℥vi

M. Et. ft. sol.

Sig. Dessert spoonful every three hours.

At 3 P. M. found patient suffering greatly from nausea; pulse 130; respiration 26; tongue presenting same appearance as in first day of illness. No change in color of skin. Again ordered patient to drink hot water, re-applied bottles of hot water, administered one-fourth gr. sulphate morphia hypoderimically, and continued treatment as adopted in forenoon.

At 9 P. M. visited our patient and found him doing well, more quiet, suffering less from nausea, pulse 100, respiration 24, tongue moist and not so red at edges and tip. Had passed about ten ounces urine, color not so wine-like, but otherwise unchanged. evidently better. Again administered morphia hypodermically and retired.

A 8 A. M. next day patient decidedly improved. Had gone through the night without distress—with but little or no nausea. He slept well, pulse 80, respiration normal, and still sweating profusely. Ordered an occasional brandy toddy, three-grain doses quinine every four hours, with iron and acid as before.

At 3 P. M. doing well. Skin a shade lighter. Urine somewhat clearer and brighter.

At 9 P. M. no perceptible change. Ordered treatment continued, and to insure a pleasant night's rest, administered one-fourth grain sulph. morphia hypodermically.

At 12 M. next day patient decidedly better. No nausea, skin and urine clearing up. No thirst, and expresses hope of speedy recovery. The only complaint now is from weakness.

We now strip him of his wet linen, change his bedding, order chicken broth, and an occasional milk punch, and advise perfect quiet for a few hours.

At 10 P. M. doing nicely, perspiring moderately, skin and urine steadily clearing up. Pulse and respiration normal, tongue almost entirely relieved of coat, pale, moist and soft. Ordered three-grain doses quinine every four hours, with chicken broth and milk punch *ad lib.*

Again visited him at 9 A. M. next day. After a quiet and peaceful night, he feels much improved and refreshed. Skin and urine rapidly clearing up. Continued quinine. Directed chicken broth and milk punch *ad lib.*

At 10 A. M. next day our patient considered out of danger, skin and urine entirely cleared up. Some appetite, gaining strength rapidly. Up to this time our patient had no action from bowels. We order now:

R. Ol. ricini.......................................℥ss
Tr. opii.......................................m. vi

To be repeated if necessary in six hours. Directed quinine in three grain doses every six hours. Continued broth and milk punch.

Visited him again at 9 P. M., he has just had a few bilious discharges from bowels—watery and quite offensive. Pulse considerably accelerated and slight nausea. We regret having directed oil, and order:

R Tr. catechu.......................................f ʒi
Tr. opii.......................................m. xv
Spts. lemon.......................................m. x
Simple syrup.......................................f ℥ss
M. Sig. At once.

Returned at 8 A. M. next day. This morning we find patient comfortable. Free from nausea, generally better, cheerful, and with a ravenous appetite. Directed quinine continued for several days, advising extreme caution as to diet, and dismissed the case.

I desire to state here that we placed upon the tongue of our patient 1 grain hydrag. chlor. mite. every morning till this time, and that he is just now slightly ptyalized.

I cannot close this communication without calling the attention of my professional brethren to the marked character of the discharges through the skin, and to its unmistakably urinous odor.

**The Opium Habit, its Successful Treatment by the Avena Sativa (?)**—The remedy to which Dr. Sell ascribes such remarkable properties (New York Med. Gaz. April 22) is a tincture of common oats. He begins by telling us that it is a very important grain, and then gives us a long history of its cultivation and uses from the time of Pliny to the present day. He discourses, too, at some length on the value of water-gruel and oatmeal tea. He next tells us that in 1874 Dr. Keith "had a concentrated tincture of the avena prepared for paralysis, from the effects of which he himself suffered three years and a half, and in three weeks, having taken the avena in fifteen drop doses three or four times a day, he was not only free from paralysis, but relieved from many serious symptoms, both mental and physical"(!) The author commenced his own observations with the concentrated tincture before hearing of Dr. Keith's case. He is convinced that it is "a most useful and reliable remedy." He finds that it is "diuretic, slightly laxative, tonic, stimulant, but especially nerve-stimulant." It is said to "exert a most powerful influence upon and through the nervous system." It is "a valuable adjuvant of other medicines," is unsurpassed in "female diseases," is "an excellent substitute for intoxicating drinks," "will cure inebriety," is "an antidote to opium-poisining"(!) It gives relief in insomnia, and is curative in nervous headache and prostration due to mental strain and worry, and in neuralgia and hemiplegia. "Epilepsy has been brought under subjection by it more effectively than by other remedies," and it is unequalled in the treatment of "hysteria, melancholia, neurasthenia, and all forms of nervous prostration, when caused by inebriety, the abuse of tobocco, opium, or morphine, by sexual excesses; masturbation, or mental strain." But this is not all, for the author has made what he describes as "a no small discovery in therapeutics." He finds that this remedy as an absolute cure for the opium or morphia habit. He gives details of three cases of morphia-taking, all of which were promptly cured by this remedy. The first patient, a German, of middle age, usually took hypodermically in the twenty-four hours from 12 to 48 or 50 grains of morphia, which had no other effect than to produce fifteen minutes sleep with the eyes wide open. The next patient, a middle aged lady, had been a slave of the morphia habit for seven years, and usually took 12 grains a day. In the third case morphia had been taken to excess for twenty years, the average being an ounce in fifteen days, or 32 grains a day. The avena in all cases works wonders. The patients are not only able to relinquish the habit, but improve in weight, strength, spirits, and mental capacity. One lady not only gained twenty-five pounds in weight in an incredibly short time, but said she felt twenty years younger. It is stated in the most positive terms that the preparation is nothing but a tincture of common oats. The dose is to be increased till it produces "the desired effect," and is to be given in hot water, "with the same frequency that the patient was accustomad to take his opium or morphia." The author displays great originality, and his powers of imagination are remarkable.—William Murrell, M. D., in The London Medical Record, 1882.—*American Medical Weekly.*

**Physiological and Therapeutical Effects of Quassine.**—Dr. Campardon, having for many years employed quassia amara in its different preparations, concluded that it had properties which ne had not seen accorded to it by others. He then made a series of experiments with amorphous quassine, and afterwards with crystalized quassin (Duquesnel), and determined that they had the same effects, and that they represent the physiological and therapeutic effects and are active principles of the drug, only in different degrees, the crystalized being ten times stronger than the amorphous. Without analyzing his experiments, we give the conclusions (Bull. Gen. de Therapuet) which he arrived at from numerous observations :

1. That amorphous quassine and crystalized quassine, active principles extracted from quassia amara (Surinam) and from quassia simaruba which are very evident and very constant;—viz:

2. In moderate doses this principle brings into activity and augments the secretion of the salivary glands, of the liver, of the kidneys, and perhaps of the mammary glands.

3. It excites the musculer fibres of the digestive tube, of the uropoietic apparatus, of the biliary ducts, augments the mucous secretion, and facilitates the exertion of the normal secretions.

4. In the sick, as a pure bitter tonic, this substance awakens appetite, builds up the forces, and, in conseqence of its action upon the muscular fibres of vegetable life, facilitates exertion, renders defecation easier, and aids the expulson of renal or hepatic culculi.

5. Quassine, as well as the wood of quassia amara and quassia simaruba, causes death in inferior animals, even in comparatively small doses.

6. In healthy individuals, as in the sick, it produces, in certain doses, a series of tonic effects, which recall the action of convulsive poisons.

7. Amorphous and crystalized quassine, above fifteen centigrammes (two and a half grains) for the first, and fifteen milligrammes (one-fourth grain) for the second dose, determine the following symptoms, which are only increased by elevating the dose: burning in the œsophagus, annular burning in the larynx, increasing constriction of the throat, frontal headache, especially to the right side, weight and pain in the region of the stomach, nausea, vertigo, troubles of sight, extreme agitation, febrile irritability, impossibility of continued thought, restlessness, frequent micturition, but which subsequently diminishes little by little, diarrhœic discharges, and vomiting. This is followed by spasmodic contractions of the muscles of organic and animal life, and cramps, due to tonic contractures of the muscles of the leg and thigh.

In order to combat the toxic effects of quassine, chloral internally, and chloform externally for the spasmodic contractures, have given the best and most prompt results.—*Medical Times.*

**Aunt Betsy Simpson's Case—What's the Diagnosis ?—** "My mis'ry, doctor, wuks right up from bofe my legs, an' up frough my stummick, an' den crost my bow'ls, all a shaky an' a wigwaggy! Den my right shoulder, doctor. *Law sakes!!!* Dat yer mis'ry in my right shoulder pow'ful bad sometimes. Den I has shootin' pains all up an' down de spine uv my back, dreafful! an' lumps in my flanks, an' a burnin' all over my right side, an' a roarin'! yes, honey, a awful roarin' in my head, an' de bones all loose in my head. Den I has pains in bofe shoulders, an' my insides dey workin' jes' like maggots!! an' I has a draggin' in my stummick, an' my sistum very bad. Ef you b'lieve me, doctor, dar's a patch of mis'ry in de small o' my back, an' when I stan' up 'pears like my insides dey stickin' to my spine! an' a wall'win' in my haid, an' I done got no appetite ter eat, an' 'pears like every minit I gwine frow up my insides. I cain't drink no fresh water; drinks all my water *biled!* In de night you can hear my head a roarin' an' a buzzin', an' den my bowels gits to wukin' an' you can hear 'em a crackin' an' a blabbin'! an' dey all a shakin' an' a trimblin'. Den I has a hotness in de bone o' my neck; yes, doctor, right in de bone o' my neck, an' at fuss a *pang* riz right up acrost my neck, an' riz an' *bust!!!* I knowed it wa'n't a blood-wessel, else I'd a died *sho!*"—*Ex.*

[Prof. Remans, who was called to see the above case, and who is regarded as the best diagnostician in America, in a note to the editor of one of our journals, says: "To my mind the case is a plain one; the left utero abdominal digitalis is occluded by a regurgitation of the right illio ceacal valve; this, of course inhibits all peristallic motion, closes the sphincter ani, makes a diverticulum of the duodenum, paralyzes the levator labii superioris and produces universal hyperesthesia throughout the entire animal economy."

**A New Point in the Diagnosis of Femoral Luxations.**—Dr. Treub, in reporting a case of obturator dislocation of the femur (Centrallblatt fur Chirurgie, No. 45), calls attention to the value of rectal exploration in order to ascertain the position taken by the head of the bone. In children the foramen ovale as well as the sciatic foramen is very easily examined by the forefinger when inserted into the rectum, and if the head of the femur is in either situation it may be easily felt. In adults it is available especially for the foramen ovale, by the aid of an anæsthetic and with the hand in the bowl the sciatic foramen may also be explored. The author therefore recommends either for a diagnostic or merely for a demonstrative purpose, in more or less obscure cases of obturator or sciatic luxation, that the rectal method of examination be tried.—*Medical Times.*

**Celerina.**—Prof. Bauer, College Physicians, &c., St. Louis, says: "Surgical practice does not frequently proffer the opportunity of employing nervo-tonic remedies, and therefore I am, perhaps, not competent to fully judge the therapeutic virtues of CELERINA, a compound lately introduced by J. C. Richardson, Esq., of this city. I have, however, used it, and with very satisfactory results, in at least twenty appropriate cases, and feel persuaded that it develops most happy actions, and that it deserves the attention of medical practitioners, more especially of those employed in the treatment of nervous afflictions. I shall certainly continue to test it more fully, and report my observation in due time.

